# DNA barcoding of blackflies (Diptera: Simuliidae) as a tool for species identification and detection of hidden diversity in the eastern regions of Spain

**DOI:** 10.1186/s13071-018-3046-7

**Published:** 2018-08-13

**Authors:** Ignacio Ruiz-Arrondo, Luis M. Hernández-Triana, Aleksandra Ignjatović-Ćupina, Nadya Nikolova, Javier Alfonso Garza-Hernández, Mario Alberto Rodríguez-Pérez, José A. Oteo, Anthony R. Fooks, Javier Lucientes Curdi

**Affiliations:** 1 Center for Rickettsiosis and Arthropod-Borne Diseases, Hospital Universitario San Pedro-CIBIR, Logroño, La Rioja Spain; 2 Department of Animal Pathology, Faculty of Veterinary Sciences, Universidad de Zaragoza, Zaragoza, Spain; 3 Wildlife Zoonoses and Vector-borne Diseases Research Group, Virology Department, Animal and Plant Health Agency, Addlestone, UK; 4 Faculty of Agriculture, University of Novi Sad, Novi Sad, Vojvodina Province Serbia; 5 University of Liverpool, Liverpool, UK; 6 Instituto de Ciencias Biomédicas, Universidad Autónoma de Ciudad Juárez, Ciudad Juárez, Chihuahua México; 7 Centro de Biotecnología Genómica, Instituto Politécnico Nacional, Reynosa, Tamaulipas México; 8 Department of Clinical Infection, Microbiology and Immunology, Institute of Infection and Global Health, University of Liverpool, Liverpool, UK

**Keywords:** DNA barcoding, Blackfly, Simuliidae, Cryptic diversity, Genetic distance, New records, Aragón region, Spain

## Abstract

**Background:**

Blackflies have negative impact on public and animal health due to the haematophagous habit of females. In recent times, in some regions in Spain, blackfly outbreaks are becoming more and more frequent, threatening the public health. However, there is still a paucity of data concerning the Spanish blackfly fauna. Correct identification of species is of paramount importance in order to provide correct information on species distribution, biology and behaviour, so that control measures could be implemented appropriately.

**Methods:**

Blackflies specimens (larvae, pupae, reared adults and biting females) were collected in the period 2015–2017 in and near rivers and streams from different regions in Spain. A modified Hotshot technique was used for the DNA extraction and the *cox*1 DNA barcoding region of the cytochrome *c* oxidase subunit 1 was sequenced from the specimens collected.

**Results:**

In total, we collected 239 specimens representing 22 species. Of these, six species are new records for the Aragón region: *P. tomosvaryi*, *S. bertrandi*, *S. galloprovinciale*, *S. lineatum*, *S. rubzovianum* and *S. xanthinum*. *Cox*1 DNA barcode sequences for 21 species were recovered, including four species of the genus *Prosimulium* and 17 species of the genus *Simulium* [*Boophthora* (1 species), *Eusimulium* (1 species), *Nevermannia* (4 species), *Simulium* (*s.s*.) (6 species), *Trichodagmia* (1 species) and *Wilhelmia* (4 species)]. For the first time the complete DNA barcodes for five species (*P. tomosvaryi*, *S. carthusiense*, *S. brevidens*, *S. monticola* and *S. sergenti*) were registered. Most of the specimens belonging to the same recognized species were clustered together in the neighbour-joining tree, except for *S. argyreatum, S. monticola* and *S. variegatum*. The overall genetic distance in the dataset was 0.14%. The average of the intraspecific genetic divergence within the different taxa was 1.47% (0.05–3.96%). In contrast, the interspecific divergence varied between 2.50–22.0%.

**Conclusions:**

In this study we assessed the use of the *cox*1 DNA barcoding region for the identification of species of blackflies in Spain. Our results showed that combining DNA barcoding with morphology enhanced our taxonomic rationale in identifying the blackflies in the country.

**Electronic supplementary material:**

The online version of this article (10.1186/s13071-018-3046-7) contains supplementary material, which is available to authorized users.

## Background

The family Simuliidae (Diptera) includes 26 genera and 2351 species (2335 extant and 16 fossil) [1]. The females of many blackfly species bite humans, birds and other animals due to their need of blood for full egg development [2, 3]. As a consequence of this hematophagic habit, simuliids can act as intermediate hosts of pathogens affecting the health of humans and animals worldwide [4]. In addition, blackflies can be used as water quality indicators due to the ecological demands and the role that the larvae play in rivers [5–9].

Integrated taxonomic research on the European blackfly fauna has been intensified in recent years, as demonstrated by the studies by Day [10], Day et al. [11, 12], Ilmonen et al. [13], Kúdela et al. [14, 15] and Adler et al. [16]. However, there is still a paucity of data concerning the blackfly fauna of Spain, where populations of some pest species have expanded recently and have become an emerging public and veterinary concern [17, 18]. In the city of Zaragoza with 700,000 inhabitants, public and animal health problems are evident because of the abundance of biting blackflies, which have resulted in a serious discomfort of herds of sheep and horses and an increase of more than 200% in recorded bites to humans between 2011 and 2012 [17, 19, 20].

Because of their environmental importance together with their impact on public and animal health, the correct identification of this insect group is of a fundamental importance in order to provide correct information on species distribution, biology and behaviour, so that targeted control measures could be correctly applied. However, standard method to blackfly species identification are mainly based on morphology, which typically require expert knowledge and sometimes the resolution can be poor because of the presence of hidden diversity [3, 21–24]. In this study we developed a molecular platform based on the *cox*1 DNA barcoding region in order to support the species identification of the poorly-studied blackfly fauna of Spain. Additionally, we explored the barcode variability to reveal hidden diversity by comparing the intra- and interspecific genetic divergence within the species we analyzed.

## Methods

### Source of material and morphological identification

**Fig. 1:**
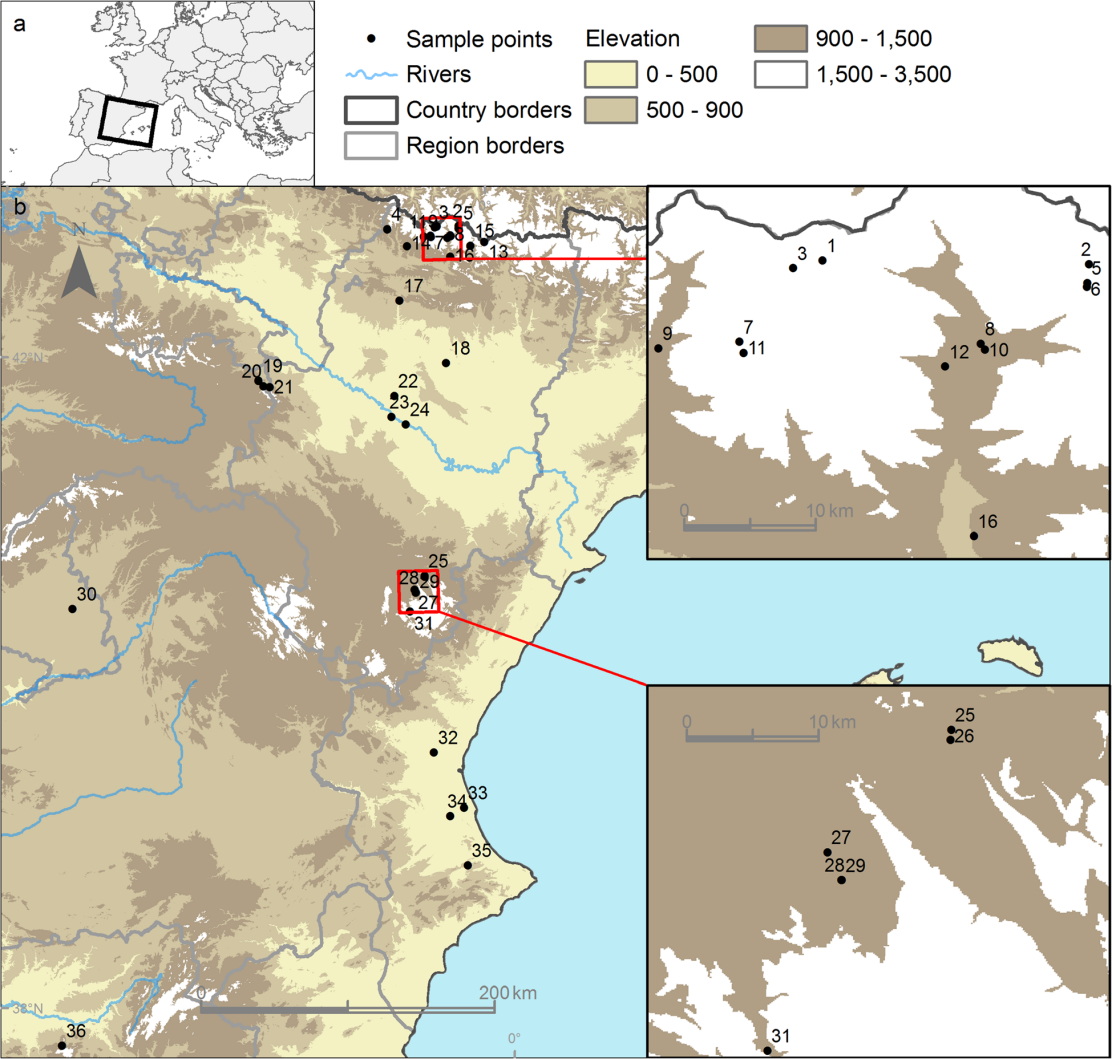
Map of the different regions in Spain, showing the localities where blackflies were collected in this study. Localities are indicated by region: Aragón: 1–18, 21–29, 31; Castile and León: 19, 20; Madrid: 30; Valencia Community: 32–35; Andalusia: 36 (for full detail regarding the exact data of the localities, the reader is referred to the BOLD project: “DNA barcoding Spanish Blackflies [SPSIM, SPSIB, SIMSP]”)

Collecting protocols established by Hernández-Triana [25] were used to collect blackflies. Larvae, pupae and link-reared adults were collected in the period 2015–2017 in rivers and streams across the Aragón region, although other areas were also surveyed (Fig. 1). Efforts were also made to collect females of species known to bite humans or livestock. Pre-imaginal specimens were preserved in 95% ethanol and held at 5 °C until molecular analysis. Adult specimens were preserved dry at -40 °C. Morphological identification of the collected material was based on descriptions given in identification keys of González [26], Bass [27] and Rivosecchi et al. [28].

### DNA extraction, PCR and sequencing

The protocols of Hernández-Triana et al. [3, 24] were used to carry out all molecular work. When pupae were selected for analysis, most of the thorax, gill, and cocoon were retained as a voucher, while the pupal abdomen and/or the thoracic region (ventral side) were used for DNA extraction. In the case of adults, only leg(s) were removed from the specimen for DNA extraction, while the remainder of the specimen was retained as a voucher. In the case of larvae, a thin layer of the integument was removed as a tissue source for analysis, while the remainder of the specimen was kept for further morphological examination.

A modified Hotshot technique from Montero-Pau et al. [29] was used for the DNA extraction. In brief, tissues were put directly into 50 μl of alkaline lysis buffer in a 96 well-plate, and sonicated in a water bath for 20 min. Subsequently, the plate was incubated in a PCR block machine for 30 min at 94 °C, and allowed to cool down for 5 min at 4 °C. Then, the plate was centrifuged for 3 min at 3000× *rpm* after which 50 μl of the neutralizing buffer was added. The plate was centrifuged again for 10 min at 3000× *rpm* and stored at -80 °C until processing the following day.

Detailed specimen records and sequence information (including trace files) were uploaded to the Barcode of Life Database (BOLD) (see http://www.boldsystems.org) and can be found within the Working Group 1.4 Initiative Human Pathogens and Zoonoses. The Digital Object Identifier (DOI) for the BOLD project is: dx.doi.org/10.5883/DS-SPSIM. All sequences have also been submitted to the GenBank database under the accession numbers MG894170-MG894340). Detailed record information can be found in the following projects in BOLD: “DNA barcoding Spanish Blackflies [SPSIM, SPSIB, SIMSP]”.

PCR amplification was carried out using the Folmer primers [30] (LCO1490 and HCO2198), which are considered the standard for the amplification of the 658 bp region located at the 5' end of the *cox*1 gene [31, 32]. PCR products were obtained using a QIAgen PCR system using the protocol of Hernández-Triana et al. [22]. We run a 1.5% agarose gel to visualize the PCR products and samples showing correct band size were sequenced in both directions using the ABI PRISM® BigDye® Terminator v3.1 Cycle Sequencing Kit (Applied Biosystems) at the Sequencing Unit, Animal and Plant Health Agency, UK.

### Sequence analysis

All bi-directional sequences were combined to produce a single consensus sequence, the full length 658 bp barcode. For certain taxonomically problematic species, the dataset also included *cox*1 sequences of some species derived from UK blackflies analyzed in Day [10] and Day et al. [12] and retrieved from the GenBank database (Table 1). We analyzed the dataset in MEGA v.6 [33], and the neighbour-joining (NJ) analysis was undertaken using the K2P distance metric to represent species distribution pattern in the NJ tree. The robustness of the NJ tree was calculated using the bootstrap methodology employing 1000 as pseudoreplicates; only groups with 70% support values were mapped in the NJ tree as suggested [33]. Barcodes longer than 500 bp were allocated a barcode index number (BIN) [34]. Each BIN was then mapped onto the NJ tree to examine their distribution among morphologically identified species. We also used the analyses capabilities of BOLD to determine the taxonomic incongruence between species identified by morphology and the *cox*1 DNA barcoding sequence [34].

**Table 1:** List of the blackfly species, country of collection, number of specimens with DNA barcodes (*n*) and mean intraspecific values of sequence divergence (%)

Species	Collection country	*n*	Mean sequence divergence (%)
*Prosimulium hirtipes*	Spain, UK	11	0.62
*Prosimulium latimucro* (*s.l.*)^a^	Spain, UK	5	2.93
*Prosimulium rufipes* (*s.l.*)	Spain	4	0.82
*Prosimulium tomosvaryi* ^a^	Spain	17	2.77
*Simulium* (*Boophthora*) *erythrocephalum*	Spain	13	0.42
*Simulium* (*Eusimulium*) *angustipes*	UK	9	1.09
*Simulium* (*Eusimulium*) *petricolum*	UK	5	1.66
*Simulium* (*Eusimulium*) *rubzovianum*^b^	Spain, UK	16	1.63
*Simulium* (*Nevermannia*) *bertrandi*	Spain	10	na^c^
*Simulium* (*Nevermannia*) *brevidens*	Spain	7	0.05
*Simulium* (*Nevermannia*) *carthusiense*	Spain	10	0.35
*Simulium* (*Nevermannia*) *cryophilum* (*s.l.*)	Spain	7	0.81
*Simulium* (*Nevermannia*) *vernum* (*s.l.*)	Spain, UK	11	0.71
*Simulium* (*Simulium*) *intermedium*^a^	Spain	21	3.96
*Simulium* (*Simulium*) *ornatum* (*s.l.*)	Spain	13	1.62
*Simulium* (*Simulium*) *argyreatum*^a^	Spain, UK	17	2.71
*Simulium* (*Simulium*) *monticola*	Spain	14	0.45
*Simulium* (*Simulium*) *variegatum*^a^	Spain, UK	22	2.15
*Simulium* (*Simulium*) *xanthinum*	Spain	1	na^c^
*Simulium (Trichodagmia) galloprovinciale*	Spain	2	0.17
*Simulium* (*Wilhelmia*) *equinum*	Spain	5	0.56
*Simulium* (*Wilhelmia*) *lineatum*	Spain	8	1.11
*Simulium* (*Wilhelmia*) *pseudequinum*	Spain, UK	6	1.19
*Simulium* (*Wilhelmia*) *sergenti*	Spain	15	0.35

^a^Taxa with high level of genetic diversity

^b^The name of *S. rubzovianum* replaced *S. velutinum* for previous distributional records of this species in Spain, which is now restricted to North Africa and Canary Islands [43]

^c^Mean intraspecific values of sequence divergence (K2P) shown with missing entries (na) indicate that less than two specimens were analyzed

## Results

**Fig. 2:**
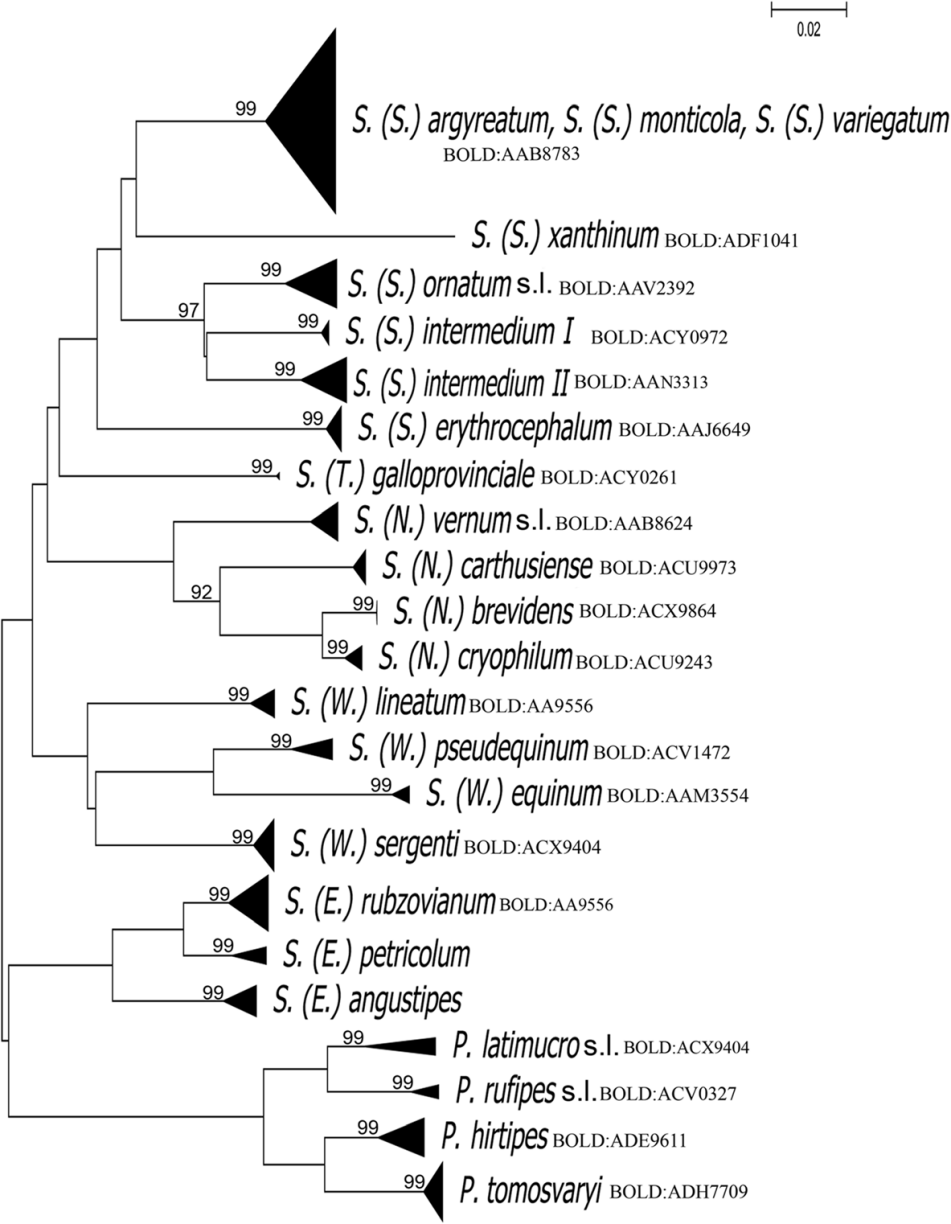
Neighbour-joining tree for species of the Simuliidae sampled in Spain based on 658 bp barcodes from the *cox*1 DNA barcoding region. Only bootstrap values higher than 70% are shown

A total of 21 morphospecies of blackflies from Spain were included in the analyses: four of the genus *Prosimulium* and 17 of the genus *Simulium*, the latter belonging to the following subgenera: *Boophthora* (1 species); *Eusimulium* (1 species); *Nevermannia* (4 species); *Simulium* (*s.s*) (6 species); *Trichodagmia* (1 species); and *Wilhelmia* (4 species) (Table 1, Fig. 2). Additionally, *S.* (*Nevermannia*) *bertrandi* was identified only morphologically, but it was not possible to obtain DNA from the single specimen we collected. Three or more representatives were available for 21 morphospecies except for *S. xanthinum* and *S. galloprovinciale* (Table 1). In total, 239 individuals were analyzed after incorporating *cox*1 sequences for certain species in Day [10] and Day et al. [12]. Of these, 225 yielded barcodes of full length 658 bp (94.14% success).

The sequence divergences varied across all taxa we analyzed. For those individuals of the same species found in the same locality the genetic diversity was zero or ranked below average values, e.g. *S. brevidens* (see Table 1). On the contrary, other individuals revealed higher values, for example *P. tomosvaryi* (Table 1). The average for the intraspecific divergence was 1.47% (range 0.05–3.96%) (see Table 1), and the average for interspecific divergence ranged between 2.50–22.0% (see Additional file 1: Table S1). All taxa belonging to separate genera (or subgenera) [1], showed increased values of genetic divergence. For example, the most divergent pairs were *P. tomosvaryi/S. equinum* (22.0%) and *P. latimucro* (*s.l.*)/*S. equinum*, *P. latimucro* (*s.l.*)*/S. monticola*, *P. tomosvaryi/S. brevidens*, *S. equinum/P. hirtipes, S. brevidens/P. hirtipes* and *S. xanthinum/P. hirtipes* (21.0%). As expected, species within the same genus or subgenus showed low genetic divergence, for example *S. brevidens/S. cryophilum* (*s.l.*) (2.5%).

In general, individuals belonging to the same species clustered together including those specimens that were collected far apart. However, not all morphospecies displayed a similar pattern (see Fig. 2). In this case, specimens recognized as *S. argyreatum*, *S. monticola* and *S. variegatum* assembled together in the same group with high bootstrap values (Fig. 2).

In this study, five species are known species complexes [1]: *P. latimucro* (*s.l.*), *P. rufipes* (*s.l.*), *S. ornatum* (*s.l.*), *S. cryophilum* (*s.l.*) and *S. vernum* (*s.l.*)*.*

The BIN calculation in the dataset of 171 barcode records was of 21 BINs, which were representatives of 21 species. Our BIN count did not include sequences from Day [10] and Day et al. [12]. In general, 133 barcodes were assigned a BIN number, which represented 16 concordant BINs, four singletons, and one discordant BIN (33 records). The discordant BIN was discordant at the species level *S. argyreatum*, *S. monticola* and *S. variegatum* (BIN AAB8783). In contrast, BIN splits were detected in *P. latimucro* (*s.l.*) (BINs ADE9519, ACX9973) and *S. intermedium* (BINs AAN3313, AAV2392) (Fig. 2).

## Discussion

Recent arguments on the utility of *cox*1 DNA barcoding in blackflies have been discussed by [22–24, 35]. In our study, known species clustered together in the NJ tree based upon *cox*1 DNA barcode sequences (Fig. 2), which demonstrate the utility of this methodology in support of species identification. Most of the individuals of a given species were correctly placed in the NJ tree. Nonetheless, morphological specimens identified as *S. argyreatum*, *S. monticola* and *S. variegatum* were mapped in the same cluster, implying that they might be conspecific. This result was not surprising as the adults of the three species are morphologically very similar. However, the three taxa can be readily identified based on the pupal gill configuration. *Simulium variegatum* is easily identified by having 1+1 prominent tubercles at the base of the gill [26], while the tubercles are absent in *S. argyreatum* and *S. monticola*. In *S. monticola*, the ventral gill filaments originate directly from the base, all filaments are prominently curved at mid length, and the cephalothorax is covered by areas of small tubercles [26]. In *S. argyreatum*, the gill is covered by tubercles, which are homogeneously distributed [26]. Thus, we advocate that different genetic markers such as the elongator complex protein 1 gene (*ECP1*) or ITS2 [36–39] should be used to explore their taxonomic status.

We expected higher levels of genetic variation between members of known species complexes, even though cytological studies were not carried out in our study [3, 22–24, 40]. With this regard, most of the specimens grouped together, and high levels of genetic diversity was not identified between species complexes. In addition, no deep divisions in the NJ tree as observed in previous studies [3, 22, 24]. This is likely due to the fact that most of the specimens originated from the same, or relatively close, localities. However, not all known species grouped as we anticipated. As a whole, we revealed high intraspecific genetic divergence not only in *P. latimucro* (*s.l.*) with 2.77%, but also in *P. tomosvaryi* with 2.93% and *S. intermedium* with 3.96%. In particular, *S. intermedium* was split into two distinct groups, named here I and II (Fig. 2). This could be indicative of the presence of a species complex, but further cytotaxonomic studies are required to validate this hypothesis. In this study, the values obtained for the intraspecific genetic divergences as well as for the interspecific genetic divergences are within the values obtained by other authors [22–24, 40–42].

Many authors (e.g. [34, 35]) have stated that the congruence found between morphologically recognized species and BINs could demonstrate the presence of cryptic genetic diversity. Therefore, the subgroups detected in *S. intermedium* may be indicative of such diversity. In contrast, the presence of same BINs in other recognized species such as *S. argyreatum*, *S. monticola* and *S. variegatum*, might be hard to explain. Therefore, we advocate for further biosystematic studies in these taxa not only in Spain, but across their distribution range.

## Conclusions

Our study augments the available information with regards to the utility of the *cox*1 DNA barcoding to assist the identification in the understudied Spanish blackfly fauna. Our results emphasize the need for continuing research using an integrated research approach using a combination of morphological traits and molecular markers, not only on the Spanish Simuliidae fauna, but also across the world. This comprehensive approach would support the correct identification of blackfly species, which in turn would have a direct effect for the implementation of the correct vector control strategies or the development of accurate protocols for studies in disease dissemination.

## Additional file

Additional file 1: **Table S1.** Interspecific (between groups) pairwise K2P genetic divergence of unique DNA barcodes, representing 23 species of the Simuliidae. (DOCX 25 kb)

## Abbreviations

BOLD: Barcode of Life Database
BIN: barcode index number
*cox*1: cytochrome *c* oxidase subunit 1
ECP1: Elongator complex protein 1
NJ tree: neighbour-joining tree
