# Supplementary material for: DNA barcoding of blackflies (Diptera: Simuliidae) as a tool for species identification and detection of hidden diversity in the eastern regions of Spain
Source: Parasit Vectors. 2018 Aug 13;11:463. doi: 10.1186/s13071-018-3046-7 (PMC6090827; doi:10.1186/s13071-018-3046-7)
Supplement: Supplementary file 1 — Table S1. Interspecific (between groups) pairwise K2P genetic divergence of unique DNA barcodes, representing 23 species of the Simuliidae. (DOCX 25 kb) [file 13071_2018_3046_MOESM1_ESM.docx]

**Additional file 1: Table S1.** Interspecific (between groups) pairwise K2P genetic divergence of unique DNA barcodes, representing 23 species of the Simuliidae. (PDF 181 kb)

|  | *latim* | *tomos* | *varieg* | *eryth* | *equinu* | *rubz* | *serge* | *inter* | *gallo* | *linea* | *pseud* | *rufip* | *brevi* | *ornat* | *carth* | *monti* | *argyr* | *xanth* | *vernu* | *cryop* | *hirti* | *angus* |
| --- | --- | --- | --- | --- | --- | --- | --- | --- | --- | --- | --- | --- | --- | --- | --- | --- | --- | --- | --- | --- | --- | --- |
| *P. latimucro* (*s.l.*) |  |  |  |  |  |  |  |  |  |  |  |  |  |  |  |  |  |  |  |  |  |  |
| *P. tomosvaryi* | 0.10 |  |  |  |  |  |  |  |  |  |  |  |  |  |  |  |  |  |  |  |  |  |
| *S. variegatum* | 0.20 | 0.19 |  |  |  |  |  |  |  |  |  |  |  |  |  |  |  |  |  |  |  |  |
| *S. erythrocephalum* | 0.19 | 0.19 | 0.13 |  |  |  |  |  |  |  |  |  |  |  |  |  |  |  |  |  |  |  |
| *S. equinum* | 0.21 | 0.22 | 0.18 | 0.18 |  |  |  |  |  |  |  |  |  |  |  |  |  |  |  |  |  |  |
| *S. rubzovianum* | 0.17 | 0.18 | 0.16 | 0.16 | 0.19 |  |  |  |  |  |  |  |  |  |  |  |  |  |  |  |  |  |
| *S. sergenti* | 0.18 | 0.19 | 0.15 | 0.15 | 0.13 | 0.16 |  |  |  |  |  |  |  |  |  |  |  |  |  |  |  |  |
| *S. intermedium* | 0.17 | 0.16 | 0.12 | 0.14 | 0.19 | 0.17 | 0.14 |  |  |  |  |  |  |  |  |  |  |  |  |  |  |  |
| *S. galloprovinciale* | 0.18 | 0.18 | 0.13 | 0.13 | 0.18 | 0.13 | 0.13 | 0.13 |  |  |  |  |  |  |  |  |  |  |  |  |  |  |
| *S. lineatum* | 0.15 | 0.16 | 0.15 | 0.15 | 0.12 | 0.16 | 0.11 | 0.16 | 0.14 |  |  |  |  |  |  |  |  |  |  |  |  |  |
| *S. pseudequinum* | 0.18 | 0.20 | 0.16 | 0.17 | 0.08 | 0.17 | 0.11 | 0.17 | 0.16 | 0.12 |  |  |  |  |  |  |  |  |  |  |  |  |
| *P. rufipes* (*s.l.*)*.* | 0.06 | 0.10 | 0.20 | 0.19 | 0.20 | 0.18 | 0.18 | 0.17 | 0.18 | 0.16 | 0.19 |  |  |  |  |  |  |  |  |  |  |  |
| *S. brevidens* | 0.19 | 0.21 | 0.16 | 0.17 | 0.19 | 0.19 | 0.17 | 0.15 | 0.16 | 0.17 | 0.17 | 0.19 |  |  |  |  |  |  |  |  |  |  |
| *S. ornatum* (*s.l.*) | 0.17 | 0.16 | 0.12 | 0.14 | 0.18 | 0.15 | 0.17 | 0.07 | 0.13 | 0.17 | 0.17 | 0.16 | 0.16 |  |  |  |  |  |  |  |  |  |
| *S. carthusiense* | 0.17 | 0.19 | 0.16 | 0.17 | 0.18 | 0.18 | 0.16 | 0.15 | 0.15 | 0.16 | 0.17 | 0.17 | 0.08 | 0.15 |  |  |  |  |  |  |  |  |
| *S. monticola* | 0.21 | 0.20 | 0.02 | 0.12 | 0.18 | 0.17 | 0.15 | 0.12 | 0.14 | 0.15 | 0.16 | 0.20 | 0.16 | 0.13 | 0.17 |  |  |  |  |  |  |  |
| *S. argyreatum* | 0.20 | 0.19 | 0.02 | 0.13 | 0.18 | 0.16 | 0.15 | 0.12 | 0.13 | 0.15 | 0.16 | 0.20 | 0.16 | 0.12 | 0.16 | 0.03 |  |  |  |  |  |  |
| *S. xanthinum* | 0.20 | 0.19 | 0.14 | 0.17 | 0.19 | 0.20 | 0.18 | 0.15 | 0.17 | 0.18 | 0.19 | 0.20 | 0.18 | 0.15 | 0.19 | 0.15 | 0.14 |  |  |  |  |  |
| *S. vernum* (*s.l.*) | 0.17 | 0.19 | 0.15 | 0.16 | 0.17 | 0.17 | 0.16 | 0.14 | 0.14 | 0.14 | 0.17 | 0.16 | 0.09 | 0.14 | 0.10 | 0.16 | 0.16 | 0.19 |  |  |  |  |
| *S. cryophilum* (*s.l.*) | 0.19 | 0.20 | 0.16 | 0.17 | 0.19 | 0.18 | 0.16 | 0.15 | 0.16 | 0.17 | 0.16 | 0.19 | 0.02 | 0.15 | 0.08 | 0.16 | 0.16 | 0.18 | 0.10 |  |  |  |
| *P. hirtipes* | 0.09 | 0.06 | 0.20 | 0.19 | 0.21 | 0.17 | 0.18 | 0.16 | 0.17 | 0.16 | 0.19 | 0.09 | 0.21 | 0.15 | 0.18 | 0.20 | 0.19 | 0.21 | 0.18 | 0.20 |  |  |
| *S. angustipes* | 0.19 | 0.19 | 0.16 | 0.16 | 0.18 | 0.08 | 0.16 | 0.16 | 0.13 | 0.17 | 0.18 | 0.19 | 0.20 | 0.15 | 0.18 | 0.16 | 0.16 | 0.20 | 0.18 | 0.19 | 0.18 |  |
| *S. petricolum* | 0.16 | 0.17 | 0.17 | 0.16 | 0.19 | 0.05 | 0.16 | 0.17 | 0.13 | 0.16 | 0.17 | 0.16 | 0.19 | 0.15 | 0.18 | 0.17 | 0.17 | 0.20 | 0.16 | 0.19 | 0.16 | 0.09 |
